# Peace, Rude Boreas

**Published:** 1868-08

**Authors:** 


### " PEACE, RUDE BOREAS."

EDITORS DENTAL REGISTER.—*Gentlemen:* — It will, ere this reaches you, have been marked, by those of your readers having the requisite intelligence, that the comments in your journal, upon my remarks on our knowledge of Dental tissues, entirely misapprehend, or else misrepresent,—intentionally misrepresent,—their character. I can not, I confess, see otherwise than the latter, for my meaning was plain, —very plain.

For example, I am represented as meaning that "connective tissue performs, in this instance, a vicarious function, that of conveying sensibility." What is this, but a misrepresentation, and an intentional one too, when you will see that the obvious meaning of my remarks, prior to and succeeding, reference to this point, were exactly the converse of this representation, since they disclaimed the sensibility, (*attributed* to these fibres, on the supposition that they are nerve fibres,) *because* they were fibres of connective tissue. How then, in stating that the latter were *not* nerve fibres, but *were* fibres of connective tissue, which are not known to convey sensibility; did I wish it understood that connective tissue conveyed sensibility? I did not, and no one so *understood* me, I am confident.

Further, I said (and the misrepresentation includes this,) that we may "imagine the dentinal fibrils, &c.," and hereupon it is emphatically represented, that I maintained it as a fact, that the dentinal fibrils do convey sensibility.

It is plain that not only was this *not* my meaning, but the very language itself, can not be represented as conveying what it is said to,—except, indeed, by intentional misrepresentation, *i. e.* by purposely assigning it a meaning it does not convey of itself.

In my language there was *no affirmation* of the claim, or any claim, such as that I am said to have made, namely, the claim that dentinal fibrils embody the function of sensibility, for I never made any claim which identified the function of sensibility with what had been observed in the dentine, but, on the contrary, throughout stated *our lack of knowledge* of what particular element of the tooth sensibility inhered in. Still I am represented as specifically affirming (or "wishing it understood,") that dentinal fibrils convey sensibility. And this when I am fully pursuaded that *only* nerve fibres do this. I said these fibres were not nerve fibres, and said this as a datum of *knowledge*, the while believing that only nerve fibres conveyed sensibility.

What was my language? "We *may imagine*." This language means, to any person in an honest mood, precisely what I *did not admit* as a *fact*, and yet from it I am represented as stating that what I thought (and so said) was imaginary or "imagined" is affirmed as a fact. And not only did I mean, to speak in this language, of what was "imagined" and said so, so precluding misapprehension, (I conceive your contributor does not *misapprehend* my language,) but the rest of my language of continuous import, leaves not the slightest room for any honest supposition that I did not have in my own mind (and embody it too in my words,) a sense of the contradistinction or controversy between what we "may imagine" and what we know. The latter only is what I asserted.

What was the rest of my language? What but this? "We are not left to imagine," &c., &c., that "they (dentinal fibrils) are nerve fibrils." Hereby plainly enough meaning, and that *broadly* too, that in the other point I had referred to, I meant and *intended* to mean, that we *were left to* imagine, *merely* imagine;—and so I had said "we may imagine the dentinal fibrils to embody the function of sensibility." Thus having the distinction broad enough between attributing sensibility to dentinal fibrils, (in reference to which point the latter two words were used,) which was something we might or "may imagine," and the knowledge of the fact that these fibrils were *not* nerve fibres, which I said we were not "left to imagine," it being a fact.

This is now plain as it was before. I *said* we "may imagine." And I said so *because* we *had* imagined this, and *were* imagining it, in imagining that connective tissue was nerve fibre, or that the dentinal fibrils were nerve fibres.

How could I have attributed sensibility to dentinal fibrils, (connective tissue,) *mistaken as* nerve fibres, when in denying that they were nerve fibres I stated them to be a tissue, which I neither thought or believed, to convey sensibility?

One point more. It is represented that I do "not pretend to inform the reader how these sentient impressions are carried from the diseased dentine to the nerves of the pulp, and how there impressed."

Well indeed, why should I *pretend* to do this? Why indeed? Except to follow the habitual example this writer sets. Is this want of pretension on my part a source of grievence to him? If I had pretended, with him, that connective tissue were nerve fibres I might have been exempt from his misrepresentation.

Why should I have pretended this when my remarks are set *against* such pretension? My remarks did not, of course, anywhere embody this or similar *pretensions*, but, on the exact contrary, set forth the true character of such pretensions and distinguished them from knowledge. If I had done what he represents me as saying, namely, that the "dentinal fibrils embody the function of sensibility for the tooth," or as he represents, really myself claimed what I said might or may be imagined, namely, "that the connective tissue performs in this instance a *vicarous* function, that of conveying sensibility from the dentine to the pulp connective tissue," then, indeed, I *should* have pretended to do what he charges me with *not doing*.

But I need say no more, for all the misrepresentations which constitute the article are similar to this one.

It is, indeed, apparent that the whole meaning and intent of my remarks were not to follow the example of this writer, and claim as known what is imaginary, for they were, to the exact contrary, a confession of the general destitution of knowledge, and so prompting us to get it. Nothing could have been more pointed than my own confession, thereof, for my own part, and to this I doubt not, all who heard the remarks referred to, will bear one and the same testimony. And is not this, in your view, the unconfessed difference between me and my commentator. I represented in my remarks that defect of knowledge, and confessed it. I disclaimed. *He*, on the contrary, pretends.

And here is the *gravamen* of my offence. I did not confess that nerve fibres had been found in the dentine, but said that extensions of connective tissue had. He claims, and what is more, insists most peremptorily in enforcing that claim upon us, very much, I judge, from the specimen I have before me, in the way one person bullies another out of his convictions that he has discovered twenty or more millions of nerves in the dentine. (See his statement.) The accuracy of this count is convincing.

I said that great observers and many of them had long and diligently sought for previously supposed nerve fibres in the dentine and had failed to find them, but that instead thereof, some of them had found connective tissue, or a form of extension of it; while he asserts that instead of this connective tissue thus seen he has discovered (and sees) millions of nerve fibres.

I made not the slightest claim to *discovery*, nor to have seen what others had not, but he claims to have seen what necessarily wholly discredits *and denies* their observations. It is true that several observers have claimed that nerve fibres exist in dentine, and thereupon I said that I could not persuade myself that any observer who had made this claim has ever seen a single nerve fibre under the microscope. This, of course, was no imputation on any of those latter observers, simply because, as Dr. Beale has shown, observers have habitually mistaken as *single* nerve fibre what turns out to be, observed under much higher powers of the microscope, leashes or compound nerves.

What can be more palpable as a fact than the one thus stated, and what less of an imputation on any observer. Since it recognizes the fact that, under high powers of the instrument, tracks of *nerves* are seen which, under low powers, appear as a single nerve. Even this the modest commentator in question, speaking of himself, says: ("a hint at myself.") Not so. This *statement of a fact has no reference to any particular observer*. And what other observer in the wide world is there who would have regarded it as an imputation on him, except one inspired by the *animus* of my critic.

Nor is there the least *likelihood* that it had. *Certainly* not to my commentator. *Is* it likely that I should have been moved to any remark as to the theory of one who held the harmless vagary that there are scores of millions of nerve fibres in dentine, and pretends he has seen them too. The problem is of very easy solution by any intelligent reader. Let him measure the *size* of dentinal substance, *i. e.* the tooth, within which it is believed those supposed nerve fibres are contained, and divide this measure by twenty or more millions and we find this belief to lead to the conclusion that about the 1,000,000 of an inch of nerve substance has been observed by my critic.

It is, indeed, very unlikely that I should have taken any notice of the holder of this fancy. "For calling them all fools" is his imputation, referring to the fellows of the *Odontographic Society.*

It appears to me that the estimation of professional brethren, in these words he assigns to me, is *not* mine but his own.

We are justified, *en passant*, in thinking so, from the fact that he is endeavoring to impose upon them the pretension that he has seen millions of nerve fibres where the best of observers have failed to find them. Where, indeed, they have found a different tissue. The character of my estimation of the Dentists, to whom my remarks were made, *testifies* to my regarding them, then and now, as intelligent, thinking men, and men having some knowledge of the subject I treated. Certainly not as men, who from lack of intelligence, could be imposed upon to accredit the assertion that any one had discovered millions of nerve fibres in dentine.

Certainly this a very low imputation, which is about as low a thing as a man can do. I venture to draw his attention to one of the ten commandments, or, indeed, the whole of them for that matter.

Of course these are but a few of the misrepresentations I am subjected to.

It is observable too that he has not, in one instance, *quoted* my *language*,—has carefully avoided that, but instead thereof, paraphrases it. This beside the broad provisions of my meanings. Thus in paraphrasing my language he represents me as having said, "*fibrils of nerves* have never been traced."

There are no such things as "fibrils of nerves" in the nomenclature of histology.

Again he says, I " found instead of a nerve *fibril.*"

Again, that "I should wish it understood that connective tissue performs a *vicarious* function," as if this nonsense and misapplication of terms of his were really mine. Again, that I have said "dentine is dead tissue," and then again associates several of these *mis*representations, under this phraseology, as what I said.

"Now here is dead tissue, with connective tissue fibrils conveying sensation to a nutrient nerve." Neither of these things did I say or mean, and of course the writer of them is aware I did not. And thereupon ask, " what congruity is there in such *arguments.*" After perpetrating this gross wrong he puts it to me thus:

" Now if connective tissue can convey sentient impressions, and has *fibrillous* structure, why not admit *them* (connective tissue is the "*them*,") to be nerves even if *they* (connective tissue is the "*they*,") are somewhat different, in minute structure, from some other nerve filaments."

This certainly is the most extraordinary display of intelligence of histological subjects that it is possible for me to conceive. I, for one, can not imagine it to be excelled in its kind by any one be he even the extremest of the class, he charged me with calling my hearers. He here means and says that, he having "represented me as saying that connective tissue can convey sentient impressions, and as having *fibrillous* structure, (which I did *not* say). I should admit *them*, *i. e.* connective tissue, to be nerves, in other words notwithstanding this is connective tissue, why not admit 'them' to be nerves?" Indeed *and* indeed, certainly, let us call them nerves, seeing that it is admitted "they are somewhat different from some other nerve filaments." ("Filaments" is correct.)

What are the characters by which we recognize nerve filaments, unless it is characters of structure, which they have in common, and by the presence of which alone we distinguish them? But the admission that the fibrils, for which the character of nerve fibre is claimed and sought, is valuable *because* it is unintentional. It discloses what has been *seen* and called nerve fibres. That is, "fibres somewhat different from some other nerve fibres."

We are fully confirmed, in our own conviction, that no nerve fibres have been seen in dentine, else, why should we be called upon to "admit" that something of a "somewhat different character in minute structure" are nerve fibres. The most probable truth of the case becomes apparent here.

My commentator goes on, saying:

"He ignores with one sweeping phrase all that I have written concerning the connection of nerve fibrils from the central plexus of the pulp, through pulp membrane and through the tubuli to the cement and enamel, simply *because he has not been able to trace them out.*"

Never was there a grosser misrepresentation. Never was there any such reason for my statement. What I stated was not from any such "*because*" nor any *because*. What I stated was a simple fact of almost *now* general knowledge, which was that fibrils of connective tissue had been found precisely where nerve fibres (not "*fibrils,*") had been looked for, and precisely where they have been said to be, just here, another tissue has been found. It was upon this *fact* I made my statement above grossly misrepresented, upon what *had* been found, and not at all upon what had *not* been found, nor upon any experience of my own in not finding something.

What was my statement, above misrepresented? "We are not left to imagine that they (*i. e.* the dentinal fibrils,) are nerve fibres, since it has been demonstrated that they are extensions of connective tissue."

The meaning, and more than that, the animating spirit of this statement is so entirely different from what I am represented to have said that I am forced to conceive the difference was intentional.

Again, I am even more literally misrepresented by my commentator. He represents me as saying "that these dentinal *fibrils* (again) *resemble* that kind of tissue, (*i. e.* connective tissue.) and not ordinary nerve fibrils (again) of axis, cylinder, and neurilemma." And to the above he adds that, ("I ought also to have said neurine,) as being true nerve structure."

Never was grosser want of knowledge, beside misrepresentation, exhibited. It more than confirms our worst suspicions. According to this statement, which is wholly *his*, not mine, true nerve structure consists of four elements: 1. "Neurine," which, by the way, he tells us we ought to have named, besides what he says we *did* say, (which we did *not*). 2. "Axis." 3. "Cylinder." 4. "Neurilemma." Such a curious substitute for the requisite knowledge this exhibits that we think it interesting. It is that nerve fibre consists of first, "neurine;" (It is not pretended we said this, for we are told we *ought* to have said, and it is, therefore, exclusively our commentator's.) second, of "axis;" third, of "cylinder;" fourth, of "neurillemma." And this is what represents his knowledge of nerve fibre, which, he conceives, qualifies him to claim superior information. And to claim that it is millions of nerve fibres, what others recognize are a different tissue.

And yet he has the hardihood to represent us as "saying that dentinal fibrils *resemble* that kind of tissue," *i. e.* connective tissue, when he himself had previously represented our very statement on this point to be

"It has been *demonstrated* they (*i. e.* dentinal fibrils,) are extensions of connective tissue."

Let us now ask him if, in honor and fairness, he has no remorse for these misrepresentations, or, at least, if in the presence of the character of his pretensions, thus brought to light, he still claims to have the requisite knowledge to insist on his claim of being regarded as more conversant with the tissue of the tooth than the observers we relied on for our information.

This pretension of knowing what *they* do not, respecting these reputed nerve fibres;—his assertion that they are unitedly ignorant where *they* believe themselves well-informed; ignorant of what nerve tissue is, is with them and their claims, *not* primarily with us who only represented knowledge we had in unanimity with theirs. For they have seen the tissue extending into the tubules, which he calls nerve "fibrils," and declare it to be an extension of connective tissue. Witness, above all others, Beale who has used the requisite powers to detect all the inequalities of its structure.

My sole offence is that I have affirmed what they affirm.

Ought he not, and can he not, with perfect justice to himself, disclaim to be an authority on any of these points; or, indeed, to be possessed of any accurate knowledge upon them? Let us see if he may not properly do so.

He cites certain information about connective tissue. And where does he go to get it? Has he studied it? That is, made it the subject of observation? Not at all. He is not in possession of any *such* knowledge or of any knowledge thereof. He merely cites the information from a book written some eleven years ago by my predecessor, Dr. Peaslee, in the New York Medical College. Dr. Peaslee's book was a compendium of what other observers, long prior, had reported, and confessedly contained no original observations.

His information is, therefore, what he quotes. But he may, with far less stretch of imagination, claim to be an authority on connective tissue, than on nerve fibres in dentine or elsewhere.

Lastly he misrepresents me, as much as he can, in saying that I assert "that only water and salts are found outside of the pulp membrane."

Whereas, I said nothing about "pulp membrane," nothing about outside or inside of pulp. Nor did I define the liquor which exudes from the pulp vessels, as water and salts.

This misrepresentation, which like the other, falsifies all I did say, shows his entire want of apprehension of the case; otherwise my meaning would have been perfectly apparent. What I said and meant *then* and now, was, that not the *whole* of the liquor sanguinus, but its "*watery* and saline ingredients," exuded. This statement was an exact discrimination for it excluded only the fibrin, its albumen and other animal matters, in permanent solution, constituting exactly what I named, "watery and saline matters," transuded from the vessels.

These "watery and saline ingredients" meaning thereby the liquor of the blood, exclusive of its soft solids, corpuscles and fibrin, are almost invariable expressions for this meaning with competent physiologists, he calls "water and salts."

Than this combined error and misrepresentation nothing to my mind could more pointedly prove his complete absence of knowledge on the subject.

Watery and saline ingredients of the blood issue from its vessels into all glandular tissues, and exclusively from the vessels of the kidneys. And my statement was, and my strong conviction, as a physiologist, is that the fluid which issues from the vessels of the pulp was restricted to these elements. It is glaringly evident that my critic was not aware of the distinction between "watery ingredients" of the blood and "water." So, therefore, he charges me with saying that only water and salts issue from the pulp vessels. But, apart from this, the whole assertion is intentionally a misrepresentation, for I said nothing about a pulp membrane. That is a pure figment of his, he imagines a pulp membrane, I neither do nor believe in it.

But all my previous conceptions of hardihood and possibilities of grievence combined are surpassed when I note what follows, as when he asks "who has analyzed this fluid?" Who, his question is, has *analyzed the fluid in a tooth cavity;* a cavity remember, nearly completely occupied to repletion by tissue. And so because no one answers I, he concludes that what I have said about the fluid (probably,) which *is* what I said, being of "watery and saline" materials is "based upon speculative theory alone," and not based upon "observation and demonstration."

Thus showing that he is so entirely uninformed, as in the absence of knowledge, to believe that the presence or absence of the fibrin, in the amount of interstitial fluid in a tooth pulp, can be observed, that is seen and demonstrated, for he distinctly objects to my statement because this has not been done.

Again, similar misrepresentations, and other want of any, the least, knowledge on the subject, is unmistakably evident in representing me as "maintaining that arteries terminate in veins, and hence there is no nutrition in the pulp," which is made up of "*alveolar*" tissue.

He does not show that there are systems of minute vessels in various organs, which, while capillary *in size,* are not ordinary capillaries, yet occupy the same relative situation to arteries and veins. Such minute vessels are described by Robin. Ordinary capillaries open into each other, like mere channels, in every possible direction, and at various angles. The others are excessively *minute branchings* of arteries. They exist in the lungs, in the kidneys, and in other situations and tissues. I did not deny them the *name* of capillaries, because *all* the very smallest vessels between arteries and veins throughout the organism, are generally so called without *distinction of name.* The same refers to the present size. But I made, and am sure of the exact propriety of making the distinction I did. The fact that I made this distinction itself showed that I was quite conversant with these differences of character. And yet I am represented as saying that "arteries terminate in veins." Had he known anything of the nature of the facts he would not have so disclosed his ignorance.

Referring to this we are asked,

"Can any person show where arteries terminate abruptly in veins."

He is ignorant of the established and well known fact, demonstrated by Suquet, that there are several "wheres" in the body where arteries terminate "abruptly," and without the intermediacy of capillaries in veins. This is the case in the skin of the elbow, parts of the hand and forearm, and corresponding parts in the lower extremities. But this is not the case in the tooth, nor has anybody but him said it was.

All these several exhibitions of ignorance, by my critic, might have been precluded, at least for this occasion, by a single look in an elementary treatise on physiology.

Beside this, and additional to the foregoing, he has misrepresented me on *all* other points. And on several points he has clearly misrepresented me *several* times, that is, as often as space would allow, and yet, he has the inconceivable hardihood to utter such brazen trumpery as this:

"I am writing this article in self-defence, and that of the profession at large." But in this, which he thinks shrewdness, he shows his lack of it, as if the profession was *identified* with him and his pretensions, and was not able to take care of itself if threatened with damages, and as if there was any call for defence on his part or theirs, except to be defended against his gratuitous assaults.

What other observer in the world, maintaining that there are nerves in dentine, would feel really called upon to speak in self-defence, from what I said upon this point?

It is because there is no such occasion for his remarks, that he undoubtedly feels it requisite to pretend there was. He would extremely like to have the profession be credulous enough to believe in his pretensions, and to believe that there was something in the case which needed his defense on their behalf, and not entirely, as the truth is, on behalf of his own private pretensions, but they are not so credulous. They know better, as well as he does.

Now, notwithstanding, I never uttered a word, nor gave a hint, contrary to the fact, that the tooth is vitally endowed, and has vital properties too, properties which maintain its form and structure; nor ever said a word contrary to the fact that its three soft, solid elements are in contact with nutritive elements, he has over and over again represented me as representing the tooth as "dead tissues." I simply represented, as a matter of conviction, that once built up, the dentine did not undergo nutrition, meaning thereby, did not undergo constant change of substance. This is what nutrition is, a consentaneous loss and supply of the substance of the texture—an atom of waste, and its exact equivalent coetaneously in supply.

Now, this charge, that I claimed the tooth was dead tissue, he has repeatedly arranged in a relation of contradiction to some equally false representation of *his*, not mine, and then charged the contradiction on me. After reading over his misrepresentations of me on this point, I being honest in the matter, was confounded with astonishment, on looking over what he had said about the tooth, to find that all the affirmation there is in my statement, adverse to the idea of being nutrified, had already been stated without the slightest reservation or qualification by himself.

Thus, under the head "Microscopy of the Teeth," by S. P. Cutler, he says: drawing the distinction between the bones and teeth, as respects nutrition, showing that the former in contradistinction to the latter, "are lined with a membrane which furnishes *nutrition* to them."

And, "the bones" are all of them covered, and their hollows lined with a membrane which furnishes nutrition to them, and, in case of disease or injury, aids in their restoration." Thus particular, in order that these facts might be widely discriminated from those in the case of teeth.

He goes on, "*on the contrary*, the teeth are not susceptible of any vital restoration after injury, or disease, with only a single exception—that is, of exostosis, etc., etc. And again, "*when these organs have been once fully developed they come to a stand-still state, or in other words, a state of inertia.*" Now these statements, in the very face of his parading, as an error made by me, and as an unpardonable one, that the dentine (not the whole tooth, mind you) after completion is not modified as *soft solid tissue is.*

We may well enquire, if he has no compunction—no scruples? But this is a case of "*self*-defence"—"myself" "defence," while at the same time it is a case of defense of the Dental profession. What striking candor characterizes this writer.

Once more, he misrepresents me, as before, with saying, "there is no nutrition carried on even in the soft pulps," and next with placing "a vaso, motor or nutrient nerve, that is, a nerve which *subserves nutrition*, "and not a sentient nerve" in this very pulp. That is, I supply a "nutrient" nerve to this pulp, which pulp, he charges me with saying, "there is no nutrition carried on (in) even in the soft pulp."

He thus, in the endeavor to fix contradiction on me, which I never made, becomes involved in contradictions which declare their real parentage, and show their purpose and intent. In plain words, he makes or invents assertions (which he says are mine) to contradict each other, and then attributes them to me.

A more utterly and exclusively *private* and *selfish animus* than this misrepresentation here, I cannot conceive or think possible. See, for illustration and proof, the animus of this phrase, which shows how utterly insensible he is to the question of dental tissue, as a question involving the interests of a profession—as a public question—as a something in science the truth of which is to be acquired in the scientific interests of that profession, and how entirely private and selfish his aim is.

"He ignores," he says, "with one sweeping phrase all that I have written;" while, just before, he had charged me with hinting at him, thus: "a hint at myself." But enough, let us hope and work for the day, when ignorance will not rule in the absence of knowledge. As I view it, the Dental profession have chosen their course and will pursue it. And though they may be confronted with the startling question; "The intelligent Dentists are prepared to adopt all," "and ignore all they had hitherto learnt from their reading and experience," they will exercise some discretion each for himself, in judging *what* they *have* "learnt," and what remains to be learned.

It appears to have been imagined by my critic that they, namely, the intelligent Dentists, had "learnt" that scores or more millions of nerve fibres, have been found in a tooth—that more nerve fibres by numerous millions, than can be found in any other like area of sensitive tissue, and not only that, but more, by millions than you can possibly compress within the structure they occupy by any mode of hydraulic compression yet devised. The above startling question, therefore, is obviously pressed upon them, not at all in the interests of knowledge, but of these nerve fibres and their discoverer. And so what with your *attributing* to the profession a belief in these fibres, and what with getting up an occasional "shindy" in behalf of that belief as theirs, when it is left unacknowledged—and what with constant reiteration of it as a fact, it may come to pass, that nobody disavowing it, you will have it all your own way, and get to be identified as the discoverer of these same fibres. The only thing to the contrary to be feared is that some unsophisticated chap, hearing your offer to "demonstrate" them, will take it in earnest, and ask for a look. You must, however, guard against any foolish expectation on his part to see them all at once, for in that case your "*fifteen inch field microscope*" will be insufficient to take them all in.

My critic's language in the beginning is this : "Now, he wishes it understood that this connective tissue performs a vicarious function, that of conveying sensibility from the dentine to the pulp tissue, and in some *way* which is not explained, *and* (mark the English) from this tissue to the nerves in the pulp." [What I said was that the dentinal fibrils could be traced into portions of that tissue.]

"He does not pretend to inform the reader how these dentinal impressions are carried from the *diseased* dentine to the nerve pulp. I did not speak of dentinal impressions, and of course did not claim the "how" of them, yet even this which as implicating me, he represents me as not pretending to do, is what he in the preceding paragraph substantially charges me with doing.

Who would believe that the whole of this is a gross *invention* of its writer. In order to accomplish this deception, he leaves unquoted "*verbatum et literatum*" our words. We said we may imagine the dentinal fibrils to *embody*, saying thereby that it *was* imaginary, though permitted in the *absence* of facts of knowledge; and our following words even more flatly presented this very meaning, saying in them " we are not left to imagine," in distinguishing the former case as one in which we were left to imagine. And yet, with my statement before him, the same as it is here, this writer represented me as wishing it understood, that the connective " tissue conveys sensibility " from the dentine to the connective tissue." Here employing his own crude notion that sensibility is something " conveyed " to misrepresent me instead of my meaning, he discloses the parentage of this notion.

Who would believe that we said not a word about *conveying sensibility*, our *confessed* dilemma being the want of any knowledge of the continuity of tissue (said to convey sensibility) with the nerves of the pulp, while in addition to all this display of our meaning, we placed a "nutrient" (as this writer names it) nerve in the pulp itself, so showing that we had not the remotest notion of wishing it understood that sensibility was conveyed, as we are represented. And, also, in addition to all these several forms of one meaning, we do not believe in the "conveyance of sensibility," and this is plain, also, for we said "embody," "we may imagine to embody," were our words.

The truth turns out to be then, that without especially intending it, I left out of my statement of what we knew, his claims or pretensions to knowledge; not as specially designing to do so, but simply for the reason, they were not included in the facts. And this, I need say, for it is abundantly evident, is the source of the *animus* which gives both spirit and tone to his misrepresentation. And this alone, namely, that I left out of the account as knowledge, his claims to the discovery of millions of nerve fibres in dentine, is the true source of his effrontery in reporting me as ignoring all that had been written, which, indeed, is further evident from the fact that he has not hinted, nor even showed that he for a moment thought of any writer but himself. His whole *animus*, therefore, confesses itself as a mere private pique, and not as arising from any interest in these questions as questions of science—questions of common interest.

Neither could he, if he essayed to do so, support this pretension, that I had ignored all that was written, make out that this "all" saving and excepting himself, claimed that there exist in dentine millions of nerve fibres. In the light of these truths, how brazen to represent, that I "proclaimed as eureka, the golden egg, the philosopher's stone," the knowledge I recounted, when, what I did, he so misrepresents, was merely and solely to present this knowledge, not at all as exclusively new to me, or discovered by me, but rather exclusively without any claim to discovery on my part.

Could the spirit which always rules and prompts in the entire absence of candor prompt to a greater enormity of misrepresentation. His latin may pass, but he may yet learn that what precedes it, is not intelligible English. His words "that category" proclaims a "plentiful lack," of the most ordinary ability to write English. He shows no more deficiency in this respect, however, than that of knowledge of what he is writing about, when he calls "connective tissue" "alveolar tissue" and speaks of nerve *fibrils*," and "*plexusus* of nerves," in the tooth.

Again, in the preceding paragraph, this writer represents me first, "as ignoring altogether all that has been written," and second, as presenting myself as "going to tell all about it." What a shameful misrepresentation! as I told first, (and did not ignore) what was known, and thereby showed, second, how limited our knowledge was, and did not claim to tell all about, but the express contrary. What I said was an express disclaimer of knowing or telling all about.

How shameful to so misrepresent me by these assertions; to assert, as he does, that I claimed to "be going to tell all about it," when, what I did, was precisely and exactly, and meaningly to the full contrary.

And it is the more shameful to so misrepresent me, when it is evident, (and made especially so by the character of his misrepresentation) that it is he and he alone, who claims to know all about it. And what is more, it is evident that it is precisely because he makes this profession, and precisely because I left that claim, or pretension, of his knowing all about it, unacknowledged, by proceeding to show, what the facts were to the contrary, that he is moved to assail me. And so far from his misrepresentations, that I ignored altogether what had been written, being the fact, I showed what had been written, and showed a certain part of it, or so much of it as represented the probable facts—and what they warranted—to be true; thus of course leaving out, or excluding as contradictory thereto, what he had written as it appears.

This latter is what moves or provokes him to say, without of course, the slightest deference to the truth that I set out with the broad assertion that at least the writers in the Dental profession, so far as relates to the Dental structures amount absolutely to nothing, ignoring altogether all that has heretofore been written.

If the author of these nerve fibres will enable any competent observer to see *one* nerve fibre in dentine, I will delight in doing him honor, and in applauding all his claims. He and I will smoke the pipe of scientific peace with him over his, in that event, well won laurels. Should he, however, be inclined to postpone this event indefinitely, and to continue his self-begotten quarrel, I must decline once for all his company, and recommend him to the attention of my "Lord Oxygen."

THE AUTHOR OF REMARKS ON DENTAL TISSUES.

